# Retinol Improves *In Vitro* Differentiation of Pre-Pubertal Mouse Spermatogonial Stem Cells into Sperm during the First Wave of Spermatogenesis

**DOI:** 10.1371/journal.pone.0116660

**Published:** 2015-02-25

**Authors:** Brahim Arkoun, Ludovic Dumont, Jean-Pierre Milazzo, Agathe Way, Amandine Bironneau, Julien Wils, Bertrand Macé, Nathalie Rives

**Affiliations:** 1 EA 4308 “Gametogenesis and Gamete Quality”, Reproductive Biology Laboratory—CECOS, Rouen University Hospital, Institute for Biomedical Research, University of Rouen, Rouen, France; 2 Biochemistry Laboratory, Rouen University Hospital, Institute for Biomedical Research, University of Rouen, Rouen, France; University Hospital of Münster, GERMANY

## Abstract

Testicular tissue freezing has been proposed for fertility preservation in pre-pubertal boys. Thawed frozen testicular tissue must undergo a maturation process to restore sperm production. The purpose of the current study was to evaluate the ability of retinol to improve the *in vitro* differentiation of pre-pubertal mouse spermatogonial stem cells into sperm. Testes from pre-pubertal mice, aged 2.5 and 6.5 days *post-partum*, were cultured on agarose gel at a gas-liquid interphase for 34, 38 and 60 days (D) and for 16, 30 and 36 D respectively. Assessment of basal medium (BM) supplemented with retinol (RE) alone, FSH/LH alone or a combination of both, was performed. Stereological analyses and tissue lesion scoring were performed at the culture time points indicated above. Sperm production was quantified at D30 and D34 after mechanical dissection of the testicular tissues. FSH/LH significantly increased the percentage of round spermatids at D30 and D38, when compared to BM alone. However, RE significantly increased the percentages of round but also elongated spermatids at D30 and D34. Moreover, RE significantly increased the number of spermatozoa per milligram of tissue at D30 and D34 when compared to BM. Therefore, RE improved the *in vitro* production of spermatids and spermatozoa from pre-pubertal SSCs during the first wave of spermatogenesis. The use of RE could be a useful tool for *in vitro* spermatogenesis from pre-pubertal human testicular tissue.

## Introduction

Male fertility preservation should be proposed before exposition to gonadotoxic treatment and is principally indicated before cancer treatment. In pre-pubertal boys, novel strategies are being introduced to cryopreserve spermatogonial stem cells (SSCs), such as testicular cell suspension or testicular tissue freezing [1–3]. However, frozen thawed SSCs must undergo a maturation process to restore sperm production. This process can be achieved through *in vitro* culture of isolated SSCs or organotypic culture of testicular fragments [4, 5].

In rodents, considerable progress has been made in the *in vitro* expansion of SSCs that can maintain their competence to initiate spermatogenesis. Indeed, it has been previously demonstrated that the incubation of mouse SSCs in presence of feeder cells (STO [SIM mouse embryo-derived thioguanine and ouabain resistant] or fibroblasts) or growth factors (GDNF [glial cell-line derived neurotrophic factor]) enables their long-term proliferation *in vitro* [6, 7]. After culture, mouse SSCs maintained their ability to differentiate and to generate functional spermatozoa upon transplantation in host testes of infertile mice [8, 9]. Moreover, pre-meiotic germ cells from 7 day-old mice were able to differentiate into spermatozoa when cultured with somatic cells in a soft agar culture system [10]. Recently, Sato *et al*. [11] generated functional sperm from pre-pubertal mouse testicular fragments positioned on agarose gel by replacing fetal bovine serum, used by Gohbara *et al*. [12], by Knock-out Serum Replacement (KSR). Spermatogenesis was maintained over two months, and the spermatids or spermatozoa obtained after *in vitro* maturation were used to produce healthy and reproductively competent offspring through intra-cytoplasmic sperm injection. Taken together, these data demonstrate an improvement in organ culture system over time through changes in the culture medium composition and in the germ cell microenvironment.

*In vivo*, many endocrine and paracrine factors are necessary to temporally and spatially control the complex process of spermatogenesis. Among these factors, it is well established that FSH, LH which acts *via* testosterone, and retinoic acid (RA, the biologically active form of vitamin A/retinol), are critical for the normal progression of spermatogenesis [13]. RA acts on both Sertoli cells and germ cells to promote the differentiation of undifferentiated spermatogonia as well as the onset of meiotic prophase, *via* the induction of the *Stra-8* (Stimulated by retinoic acid-8) gene during the first wave of spermatogenesis [13, 14]. Furthermore, it has been postulated that, in Vitamin A-deficient (VAD) rats, RE is required in spermatocytes during the transition to round and elongating spermatids to support the full development of spermatogenic cells into elongated spermatids [15]. RA originating from Sertoli cells is also necessary for the proper release of elongated spermatids into the lumen of the seminiferous tubules during spermiation in mice [16]. However, little is known about the role of RE or RA during the post-natal development of mice testes, particularly during the initiation of spermatogenesis in pre-pubertal mice testes. Recently, it has been demonstrated in VAD pre-pubertal mice that vitamin A appears to regulate the initiation of meiosis I during the first wave of spermatogenesis [14]. Furthermore, the RA synthesized by Sertoli cells is indispensable for initiating the differentiation of undifferentiated A-aligned spermatogonia into differentiated A1 spermatogonia during the first pre-pubertal spermatogenic cycle [16]. These data were also confirmed by the observation that mice gonocytes and undifferentiated spermatogonia obtained from cultured neonatal testes or isolated gonocytes/spermatogonia exhibited limited *in vitro* differentiation after RA stimulation in the absence of Sertoli cells [17].

Recently, our group demonstrated the beneficial effect of RE on the *in vitro* maturation of fresh and thawed frozen mouse pre-pubertal SSCs in an organ culture system using polycarbonate membrane inserts [18]. Indeed, RE at a concentration of 10^-6^ M, rather than RA, maintained intra-tubular cell proliferation, the ability of spermatogonia to enter meiosis and Leydig cells functionality [18]. However, this system of culture did not allow pachytene spermatocytes to reach the post-meiotic stage and induced significant testicular tissue necrosis after 11 days of culture [18]. Therefore, we aimed to improve the production yield of spermatozoa from pre-pubertal mice SSCs by using the agarose gel system at a gas-liquid interphase. In this study, we supplemented the culture medium with RE alone, FSH/LH alone or a combination of both. KSR, originally optimized for undifferentiated embryonic stem cell culture but now also used for *in vitro* mice spermatogenesis, does to our knowledge not contain vitamin A or any active metabolite forms of it [19].

Our results demonstrate that retinol enhanced not only the rates of round and elongated spermatid formation but also the number of spermatozoa after *in vitro* culture of pre-pubertal mice SSCs during the first wave of spermatogenesis.

## Materials and Methods

### Culture media and reagents

The basal culture medium (BM) used as a control was composed of α-Minimum Essential Medium (α-MEM; Life Technologies, Carlsbad, California, USA), 10% (v/v) KSR (Life Technologies) and gentamycin (Sigma-Aldrich, Saint-Quentin Fallavier, France) at a final concentration of 5 μg/ml. The basal medium was supplemented with FSH (Puregon; MSD France, Courbevoie, France), LH (Gonadotrophine Chorionique Endo; MSD France, Courbevoie, France) and/or RE (Retinol; Sigma Aldrich). Four media were compared: (i) BM alone (without FSH/LH or RE) (ii) BM supplemented with 500 IU/L FSH and 50 IU/L LH, (iii) BM supplemented with 10^-6^ M RE [18] and (iv) BM supplemented with 500 IU/L FSH, 50 IU/L LH and 10^-6^ M RE.

### Animals and collection of testes

All experimental procedures were approved by the Institutional Animal Care and Use Committee of Rouen University under licence number 76–120. CD-1 mice aged 2.5 and 6.5 days *post-partum* (*dpp*) were euthanised by decapitation (Charles River Breeding Laboratories, L’Arbresle, France). The testes were excised and rinsed in α-MEM. The tunica albuginea was removed using sterile needles, and the testes were then rinsed again in the medium described above. For each litter, one testis was fixed in Bouin’s solution to ensure that the seminiferous cords contained no germ cells more advanced than gonocytes (for 2.5 *dpp* mice) or spermatogonia (for 6.5 *dpp* mice). In neonatal mice, gonocytes have large spherical nuclei and are present at the centre of the seminiferous cords that could be easily identified under the microscope. From 3.5 to 5.5 *dpp*, gonocytes migrate from the centre to the peripheral region of the seminiferous tubules to become spermatogonia [20]. Therefore, at 6.5 *dpp* spermatogonia could be identified with their spherical nuclei but present on the basement membrane of the seminiferous tubules.

### Culture method

**Fig 1:**
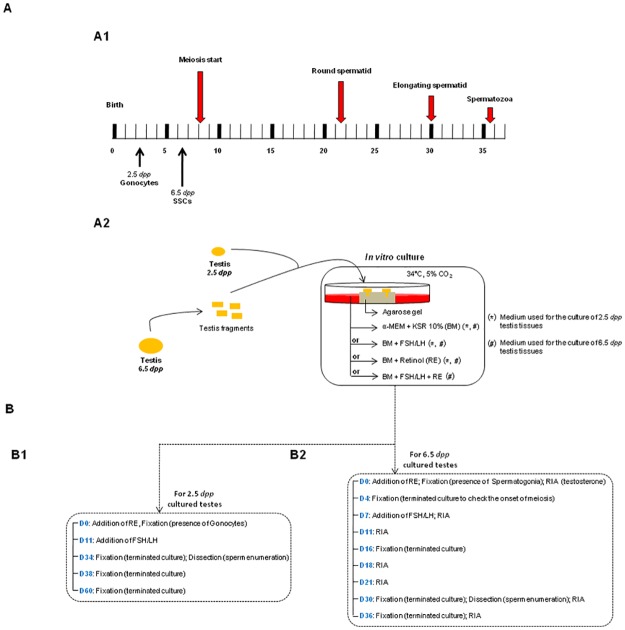
Time course of spermatogenesis in mice and i*n vitro* culture procedure. (**A**) Schematic overview of the timeline of spermatogenesis during post-natal mouse development (**A1**) and procedure for the *in vitro* culture of prepubertal mice testes used in the present study (**A2**). The culture media tested for the culture of 2.5 and 6.5 *dpp* mice testis tissues are shown with (*) and (#) symbols, respectively. (**B**) Set of experiments performed after culture of 2.5 (**B1**) and 6.5 *dpp* (**B2**) mice testis tissues. The days of culture are mentioned according to the experiment performed for each age group (**B1** and **B2**). ***Footnotes*: BM**: Basal Medium, **D**: Day, ***dpp***: day *post-partum*, **KSR**: Knock-out Serum Replacement, **FSH**: Follicle Stimulating Hormone, **LH**: Luteinizing Hormone, **α-MEM**: alpha-Minimum Essential Medium, **PAS**: Periodic Acid-Schiff, **RE**: Retinol, **RIA**: Radioimmunoassay, **SSCs**: Spermatogonial Stem Cells

A schematic overview of the time course of spermatogenesis in mice and the experimental procedure can be found in Fig. 1A1. Testicular tissues were partially inserted into small holes in 1.5% (w/v) agarose gels (A6013–10G, Sigma-Aldrich) that had been pre-soaked overnight in BM to replace water. Each agarose gel strand was 7 mm in height and 1.3 ml of the culture medium was used for each Petri dish (Center-Well Organ Culture Dish-BDFalcon; BD Biosciences, Franklin Lakes, New Jersey, USA). Two pieces of agarose gels, each containing two testicular tissues, were then placed into a small Petri dish containing the basal culture medium alone or supplemented with FSH/LH and RE as described above (see “Culture media and reagents”). The incubator used for cultures contained 5% CO_2_ and 95% air and was maintained at 34°C (Fig. 1A2).

For 2.5 *dpp* and 6.5 *dpp* mice testis tissues, RE was added to the BM beginning at day 0 (D0) of culture. In addition to RE, we also performed pilot studies using our organ culture system to test the addition of different concentrations of FSH and LH to the BM at D0, D7 or D14 of culture (S1–S3 Tables, respectively). The simultaneous addition of FSH (500 IU/L) and LH (50 IU/L) beginning at D7 of culture was selected because it induced the highest rates of spermatid formation after *in vitro* culture of 6.5 *dpp* pre-pubertal mice testes (S2 Table). FSH/LH was added to BM alone or in combination with RE beginning at day 7 (D7) of culture when 6.5 *dpp* testes tissues were cultured for 30 days. Consequently, FSH and LH were introduced simultaneously at D11 of culture when 2.5 *dpp* mice testes were used for *in vitro* maturation.

For 2.5 *dpp* pups (Fig. 1B1), whole testes of approximately 0.75 mm^3^ each, were used and partially inserted into the agarose gel to prevent their slippage. Testes were cultured for 34 (D34) and 38 (D38) days to explore specifically the end of the first wave of spermatogenesis and the onset of puberty, respectively. It was previously demonstrated that *in vitro* production of spermatids from 2.5 *dpp* mice testes decreased significantly after long term culture, notably at 60 (D60) days of culture with medium supplemented with KSR alone [11]. In this study, 2.5 *dpp* mice testes were also cultured for D60 to evaluate the effects of RE or FSH/LH on spermatogenesis efficiency during a long term culture period. For 6.5 *dpp* pups (Fig. 1B2), each testis was cut into 4 pieces of approximately 0.75 mm^3^ each, and the pieces were partially inserted into the agarose gel. Testis tissues were cultured for: i) 16 (D16) days to coincide with the end of meiosis and the apparition of the first round spermatids, ii) 30 (D30) days to correspond with the end of the first wave of spermatogenesis, and iii) 36 (D36) days to coincide with the onset of puberty in mice. Furthermore, testes explants (6.5 *dpp*) were cultured for 4 days to assess the expression of Sall-4, c-kit and Stra-8 at the onset of meiosis and therefore at the initiation of spermatogenesis in the presence or absence of RE in the culture medium.

Four testes from mice of different litters were used for each age group and culture condition tested. The culture media were changed every 3 to 4 days for each culture condition tested until the culture was terminated. Testes from [2.5-, 6.5-], 22.5-, 36.5-, 40.5-, 42.5-, and 62.5-day-old mice, corresponding to [D0] and the five different culture times (D16, D30, D36, D38 and D60, respectively) were used as *in vivo* controls.

### Histological evaluation

Cultured and *in vivo* control testicular tissues were immediately fixed in Bouin’s solution (Sigma-Aldrich) for 2 hours at room temperature and embedded in paraffin. For each specimen, three sections (3 μm) obtained at intervals of 15 μm were evaluated *via* periodic acid-Schiff (PAS)-haemalum (RAL diagnostic, Martillac, France) staining to allow a global evaluation of germ cell differentiation and seminiferous tubule architecture. Integrity and structural changes of *in vivo* and *in vitro* seminiferous tubule sections were evaluated semi-quantitatively. The seminiferous epithelium was scored as described by Milazzo *et al*. [21]: (i) detachment of cells from the basement membrane was scored as 0 if absent, 2 if partial and 3 if total or observed in more than 75% of the circumference; (ii) gap formation and shrinkage were scored as 0 if absent, 1 if slight and 2 if more obvious. Therefore, the global score for each seminiferous epithelium section was between 0 and 5. For each specimen, the mean score obtained from 30 seminiferous tubule sections was used as the global score.

### Immunohistochemistry

Tissue sections (3 μm) were stained for Sall-4 (for undifferentiated type A spermatogonia) [22], c-kit (for differentiating spermatogonia), Stra-8 (for preleptotene Spermatocytes), Tra98 (for spermatogonia as well as leptotene/zygotene and pachytene spermatocytes I), cAMP-responsive element modulator (CREM-1, for post-meiotic spermatids) and acrosin (for the acrosome of post-meiotic spermatids). Briefly, sections were deparaffinized, rehydrated in a graded ethanol series and incubated in 10 mM citrate (pH 6) (Sigma-Aldrich) for 40 minutes at 96°C. Endogenous peroxidases were inactivated by a 5 minute-treatment with hydrogen peroxide (Thermo Scientific, Massachusetts, USA), followed by the blocking of non-specific antibody binding with 5% normal horse serum (Thermo Scientific) for 10 minutes at room temperature. The slides were incubated with a primary antibody (rabbit anti-Sall-4A/B [SALL-4; 1:400, Abcam, Paris, France], goat anti-c-kit [M-14; 1:100, Santa Cruz Biotechnology, Heidelberg, Germany], rabbit anti-Stra-8 [STRA-8; 1:2000, Abcam], rat anti-Tra98 [TRA98; 1:50, Abcam] overnight at 4°C; rabbit anti-CREM-1 [X-12; 1:50, Santa Cruz Biotechnology] for 60 minutes at room temperature; or goat anti-acrosin [C-14; 1:25, Santa Cruz Biotechnology] overnight at 4°C). Negative controls were performed with pre-immune rabbit IgGs (SC-2027, Santa Cruz Biotechnology) or pre-immune goat IgGs (SC-3887, Santa Cruz Biotechnology) or rat pre-immune IgGs (SC-2026, Santa Cruz Biotechnology) according to the primary antibody used. The washing steps following the primary antibody incubation were performed using Tris-buffered saline (TBS; Dako, Paris, France).

For the detection of Sall-4, sections were incubated with biotinylated Goat anti-Polyvalent (Ultra vision Plus, Thermo scientific). Tra98 was detected with a secondary antibody (1:200; Rabbit anti-rat IgGs/HRP, Dako) and subsequently incubated with biotinylated tertiary antibody (1:200; biotinylated swine anti-rabbit IgG, Dako). Stra-8 and CREM-1 were detected using biotinylated goat anti-rabbit IgG (1:300; 1:200; respectively. Santa Cruz Biotechnology). Furthermore, c-kit and acrosin were detected using biotinylated donkey anti-goat IgG (1:200; 1:100; respectively. Santa Cruz Biotechnology). Specific staining was achieved by incubation with a streptavidin-horseradish peroxidase solution (HRP, Thermo Scientific) for 15 minutes followed by an incubation with 3,3′-diaminobenzidine substrate (DAB, Thermo scientific) for 1–10 minutes. Finally, the nuclei were counterstained with haematoxylin (Dako). The proliferative capability of the intra-tubular cells was evaluated using an anti-proliferating cell nuclear antigen antibody (PCNA, Invitrogen, Carlsbad, California, USA) according to the manufacturer’s instructions.

Furthermore, the spermatozoa present in cell suspensions obtained from cultured testicular fragments were identified by immunofluorescent staining of their flagella using a monoclonal mouse anti-acetylated tubulin antibody (T6793, 1:100, Sigma) (see “Sperm enumeration” below). A pre-immune mouse IgG (SC-2025, Santa Cruz Biotechnology) was also used as a control antibody with the same procedure. Subsequently, the spermatozoa were incubated with a FITC-labelled goat anti mouse IgG secondary antibody (F4143, 1:100, Sigma-Aldrich). The slides were mounted in medium containing 4′,6-diamidino-2-phenylindole (DAPI; Abbott, Chicago, Illinois, USA) and analyzed using an epifluorescence microscope at a ×600 magnification (BX61; Olympus, Tokyo, Japan).

### Stereology

Testicular tissue sections from each *in vitro* condition and the corresponding *in vivo* samples (see “Culture method”) were analyzed at a ×500 magnification under a light microscope (DM4000B; Leica, Solms, Germany) equipped with Leica Application Suite software (Germany). To assess the proportions of haploid spermatids in seminiferous tubules, the mean proportion of intra-tubular cells (Sertoli cells, spermatogonia, spermatocytes I, round spermatids and elongated spermatids) was determined in 30 cross-sectioned tubules in three sections obtained at intervals of 15 μm. Tra98 immunostaining, as described above (see “Immunohistochemistry”), allowed an accurate distinction between Sertoli cells (Tra98-negative cells) and germ cells (spermatogonia as well as leptotene/zygotene and pachytene spermatocytes I: Tra98-positive cells). Thus, intra-tubular cells were classified as Sertoli cells (irregular blue nuclei), spermatogonia (smooth spherical brown nuclei), leptotene/zygotene primary spermatocytes (irregular spherical brown nuclei with condensed chromatin), pachytene primary spermatocytes (irregular spherical brown nuclei with highly condensed chromatin), round spermatids (regular small round blue nuclei), or elongated spermatids (elongated blue nuclei with highly condensed chromatin). Subsequently, the mean proportion of tubule sections at the most advanced stage of spermatogenesis was determined in 30 cross-sectioned tubules in three sections obtained at intervals of 15 μm.

Furthermore, to assess the effect of RE on spermatogenesis initiation *in vitro* (at D4 of culture), the mean percentage of positive tubules for Sall-4, c-kit and Stra-8 was determined in 20 cross-sectioned tubules. Therefore, the mean percentage of undifferentiated type A spermatogonia (Sall-4 +), differentiating spermatogonia (c-kit +), and premeiotic germ cells (Stra-8 +) per cross-sectioned tubule was also determined.

The seminiferous tubule area and intra-tubular cell density (number of intra-tubular cells/1000 μm^2^) were assessed simultaneously in 10 cross-sectioned tubules using a digital imaging analysis system (LAS, Leica) connected to an inverted microscope (DM4000B, Leica). A cross-sectioned tubule was defined when a ratio of less than 1.5 was observed between the longest diameter of the tubule and the diameter perpendicular to it. Subsequently, the mean cellular density per tubule cross section was determined. The percentage of PCNA-positive cells was calculated based on the examination of at least 500 intra-tubular cells.

### Sperm enumeration

Four testes obtained from 2.5 *dpp* and 6.5 *dpp* mice from different litters were cultured for 34 and 30 days respectively, under the different culture conditions mentioned above. At the end of the culture period, each testis was weighed, placed into a Petri dish (BD Biosciences) containing 500 μl of α-MEM at 34°C with 5% CO_2_ and shredded with insulin syringes. Subsequently, 20 μL of each testicular cell suspension was observed under a light microscope at a ×400 magnification (Laborlux, Leica) to identify and count the generated spermatozoa. Thereafter, the remaining testicular cell suspension was centrifuged for 10 minutes at 600 *g* at room temperature. One portion of the pellet was subjected to Shorr staining (Merck, Darmstadt, Germany), and the other portion was fixed with methanol and stored at -20°C for acetylated tubulin immunostaining. A control testicular sperm sample from a 36.5 *dpp* old testis was performed with the same procedure for Shorr staining, for acetylated tubulin detection and sperm enumeration.

### Testosterone measurement using a radioimmunology assay

*In vitro* steroidogenesis was evaluated by measuring testosterone secretion into the medium. Media samples were stored and analyzed at: (i) D7 and D11 to coincide with meiosis progression, (ii) D18 and D21 where spermiogenesis already started, (iii) D30 to coincide with the end of the first wave of spermatogenesis and (iv) D36 to coincide with the onset of puberty. Testosterone dosage was performed using a direct radioimmunoassay (Testosterone kit (IM1087); Immunotech Beckman-Coulter Company, Roissy, France). The samples were assayed in duplicate according to the manufacturer’s recommendations. The assay had a lower limit of sensitivity of 0.04 ng/mL with average intra- and inter-assay variations of 7.2% and 10.7%, respectively.

### Statistical analysis

Statistical analysis was performed for all experiments using the Friedman test for global comparisons, the Mann-Whitney test for unpaired rank comparisons and the Wilcoxon test for paired rank comparisons. The data are presented as the means ± s.e.m. A *p* value below 0.05 was considered statistically significant. The presence of a relationship between the seminiferous tubule area and the ratio between germ cells and Sertoli cells was assessed for all the culture conditions tested by calculating the Pearson’s correlation coefficient.

## Results

### RE does not prevent seminiferous tubule growth during culture, while FSH/LH increases seminiferous tubule growth after in vitro culture of pre-pubertal mice testis. Intra-tubular cell density decreases regardless of the culture medium tested. The ratio between germ cells and Sertoli cells increases when seminiferous tubules grow in cultured 6.5 dpp old testes, regardless of the culture medium used

#### (i) Cultures of 2.5 dpp mice testes

**Fig 2:**
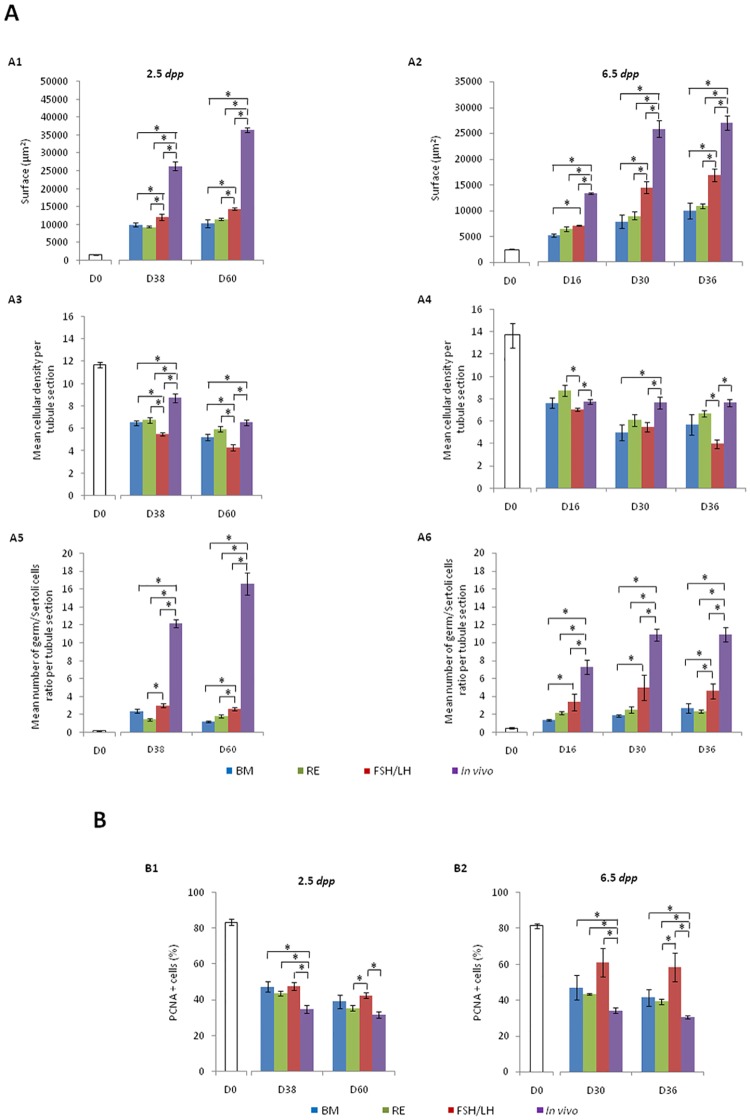
Seminiferous tubule growth (A1, A2), intra-tubular cell density (A3, A4), germ/Sertoli cells ratio (A5 and A6) and intra-tubular cell proliferation (B1, B2) during organotypic culture of prepubertal mice testes under different culture conditions. The results are presented as the mean ± s.e.m., with *n* = 4. Asterisk indicates a statistically significant difference (*p*<0.05). ***Footnotes*: BM**: Basal Medium, **D**: Day, ***dpp***: day *post-partum*, **FSH**: Follicle Stimulating Hormone, **LH**: Luteinizing Hormone, ***n***: Number of mice testes used in each condition, **PCNA**: Proliferating Cell Nuclear Antigen, **RE**: Retinol, **s.e.m.**: Standard Error of the Mean

The seminiferous tubule surface (Fig. 2A1) increased significantly during the culture for FSH/LH (p = 0.04) and RE (p = 0.0046) as observed *in vivo* (p = 0.0046), even if this surface was significantly higher in the corresponding *in vivo* controls than under *in vitro* conditions (p = 0.01). Moreover, the greatest seminiferous tubule areas were obtained with FSH/LH when compared with BM and RE at D38 (p = 0.02 and p = 0.01 respectively) and D60 (p = 0.01). However, seminiferous tubule surface was similar at D38 and D60 between BM and RE.

In contrast, while the seminiferous tubule area increased, the intra-tubular cell density (Fig. 2A3) significantly decreased during the culture for BM (p = 0.0046), FSH/LH (p = 0.0046) and RE (p = 0.04) as observed *in vivo* (p = 0.0046), even if this density was significantly higher in the corresponding *in vivo* controls than under *in vitro* conditions (p = 0.01), except for RE at D60. FSH/LH caused a lower intra-tubular cell density compared with BM (D38 [p = 0.01] and D60 [p = 0.02]) and RE (p = 0.01, for both).

The ratio between germ cells and Sertoli cells (Fig. 2A5) increased significantly during the culture for BM (p = 0.01), FSH/LH (p = 0.01), RE (p = 0.04) as detected *in vivo* (p = 0.01), even if this ratio was significantly higher in the corresponding *in vivo* controls than under *in vitro* conditions (p = 0.01). FSH/LH significantly increased the ratio between germ cells and Sertoli cells compared with BM (D60 [p = 0.01]) and RE (D38 [p = 0.02] and D60 [p = 0.01]). The mean area of seminiferous tubules and the ratio between germ cells and Sertoli cells were not correlated during the culture from D0 to D60 for BM (r = 0.84; p = 0.36), FSH/LH (r = 0.94; p = 0.21), RE (r = 0.97; p = 0.15) as observed *in vivo* (r = 0.98; p = 0.08).

#### (ii) Cultures of 6.5 dpp mice testes

The seminiferous tubule surface (Fig. 2A2) increased significantly during the culture for BM (p = 0.0009), FSH/LH (p = 0.0009), RE (p = 0.006) as observed *in vivo* (p = 0.001). FSH/LH lead to the greatest seminiferous tubule surfaces compared to BM (D16 [p = 0.01], D30 [p = 0.01] and D36 [p = 0.02]) and RE (D30 [p = 0.02] and D36 [p = 0.02]) with a time-dependent evolution following a curve comparable to that observed *in vivo* but with significantly lowest values (p = 0.01). However, RE did not modify seminiferous tubule growth or intra-tubular cell density compared with BM.

The intra-tubular cell density (Fig. 2A4) decreased significantly for BM (p = 0.0062), FSH/LH (p = 0.0001) and RE (p = 0.0009) during the culture from D0 to D36, and remained significantly lower compared to *in vivo* with BM (D30 (p = 0.02) or FSH/LH (D16 [p = 0.01], D30 [p = 0.02] and D36 [p = 0.01]).

The mean ratio between germ cells and Sertoli cells (Fig. 2A6) was significantly higher in the corresponding *in vivo* controls than under *in vitro* conditions (p = 0.01). However, FSH/LH significantly increased the ratio between germ cells and Sertoli cells when compared with BM (D16 [p = 0.01], D30 and D36 [p = 0.02]) or RE (D36 [p = 0.02]). RE had no effect on this ratio when compared with BM whatever the time point of culture tested. Furthermore, the seminiferous tubule area and the ratio between germ cells and Sertoli cells were positively and significantly correlated during the culture from D0 to D36 for BM (r = 0.98; p = 0.012), FSH/LH (r = 0.99; p = 0.041), RE (r = 0.99; p = 0.017) and *in vivo* (r = 0.98; p = 0.014).

### PCNA expression decreases during in vitro culture regardless of the culture medium tested but its evolution is close to that of the in vivo controls with RE. However, FSH/LH induces the highest PCNA expression compared to other conditions

#### Cultures of 2.5 dpp mice testes

PCNA expression (Fig. 2B1) decreased significantly from D0 to D60 for BM (p = 0.0046), FSH/LH (p = 0.04), RE (p = 0.0046) and *in vivo* (p = 0.0046). The percentage of PCNA-positive cells was significantly lower in the corresponding *in vivo* controls than under *in vitro* conditions at D38 and D60 for all tested conditions (p = 0.01) except with BM (p = 0.057) and RE (p = 0.1) at D60 of culture. FSH/LH significantly increased PCNA expression when compared with RE (p = 0.02) at D60 of culture. However, the evolution of PCNA expression during culture was close to that of the *in vivo* controls with RE.

#### Cultures of 6.5 dpp mice testes

PCNA expression decreased significantly from D0 to D36 (6.5 *dpp* mice) for BM (p = 0.0046), RE (p = 0.04) and *in vivo* (p = 0.041) with the exception of the FSH/LH condition (p = 0.069) (Fig. 2B2). The percentage of PCNA positive cells was significantly lower in the corresponding *in vivo* controls than under *in vitro* conditions at D30 and D36 for all tested conditions (p = 0.01). FSH/LH significantly increased PCNA expression when compared with RE (p = 0.02) at D36 of culture. However, the evolution of PCNA expression during culture was close to that of the *in vivo* controls with RE.

### RE, but not FSH/LH, better maintains the seminiferous epithelium integrity during in vitro culture of pre-pubertal (2.5 dpp and 6.5 dpp) mice testes

**Fig 3:**
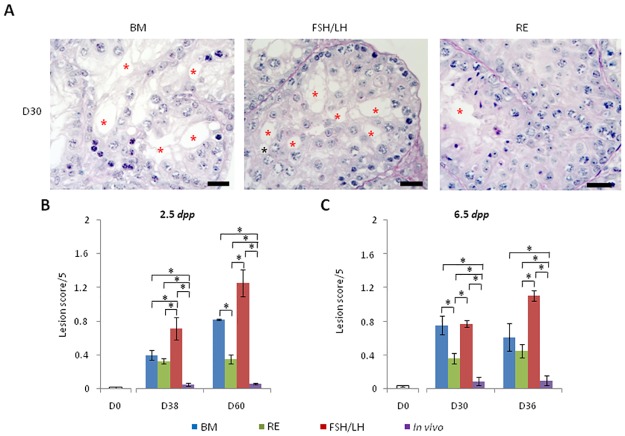
Lesion score assessment in seminiferous tubules during organotypic culture of pre-pubertal mice testicular tissue under the tested conditions. (**A**) Histological evaluation of alterations in the seminiferous epithelium after staining with PAS and haematoxylin. Photomicrographs were captured at a ×500 magnification, and the scale bar represents 20 μm. Gap formation (vacuole) is represented by red asterisks (*). (**B**) Mean global lesion score of seminiferous tubules at D0, D38 and D60 of culture (2.5 *dpp*) and **C**) D0, D30 and D36 of culture (6.5 *dpp*) for BM, RE, FSH/LH and *in vivo* conditions. The results are represented as the mean ± s.e.m., *n* = 4. Asterisk indicates a statistically significant difference with *p*<0.05. ***Footnotes*: BM**: Basal Medium, **D**: Day, ***dpp***: day *post-partum*, **FSH**: Follicle Stimulating Hormone, **LH**: Luteinizing Hormone, ***n***: Number of mice testes used in each condition, **PAS**: Periodic Acid-Schiff, **RE**: Retinol, **s.e.m.**: Standard Error of the Mean

Cell detachment and gap formation in seminiferous tubules increased significantly from D0 to D60 (Fig. 3B) regardless of the tested conditions (p = 0.0046) and from D0 to D36 (Fig. 3C) for BM (p = 0.04), FSH/LH (p = 0.0046), RE (p = 0.0046) and *in vivo* (p = 0.0046). The tissue alteration scores were significantly higher for FSH/LH when compared with BM at D36 (p = 0.02), D38 (p = 0.02) and D60 (p = 0.01). RE significantly reduced the cell detachment and gap formation of seminiferous tubules compared with BM (D30 [p = 0.02] and D60 [p = 0.01]) or FSH/LH regardless of the culture time points tested (p = 0.01) (Fig. 3A, 3C). Morphological changes in the seminiferous tubules were significantly higher *in vitro* than those observed for the corresponding *in vivo* controls at all culture time points and under all tested conditions (p = 0.01).

### RE does not prevent self-renewal of SSCs (sall-4) at D4 and their maintenance at D16 and D30 but enhances SSC differentiation (c-kit) and their entry into meiosis (Stra-8) at D4 in cultured 6.5 dpp testis tissues

**Fig 4:**
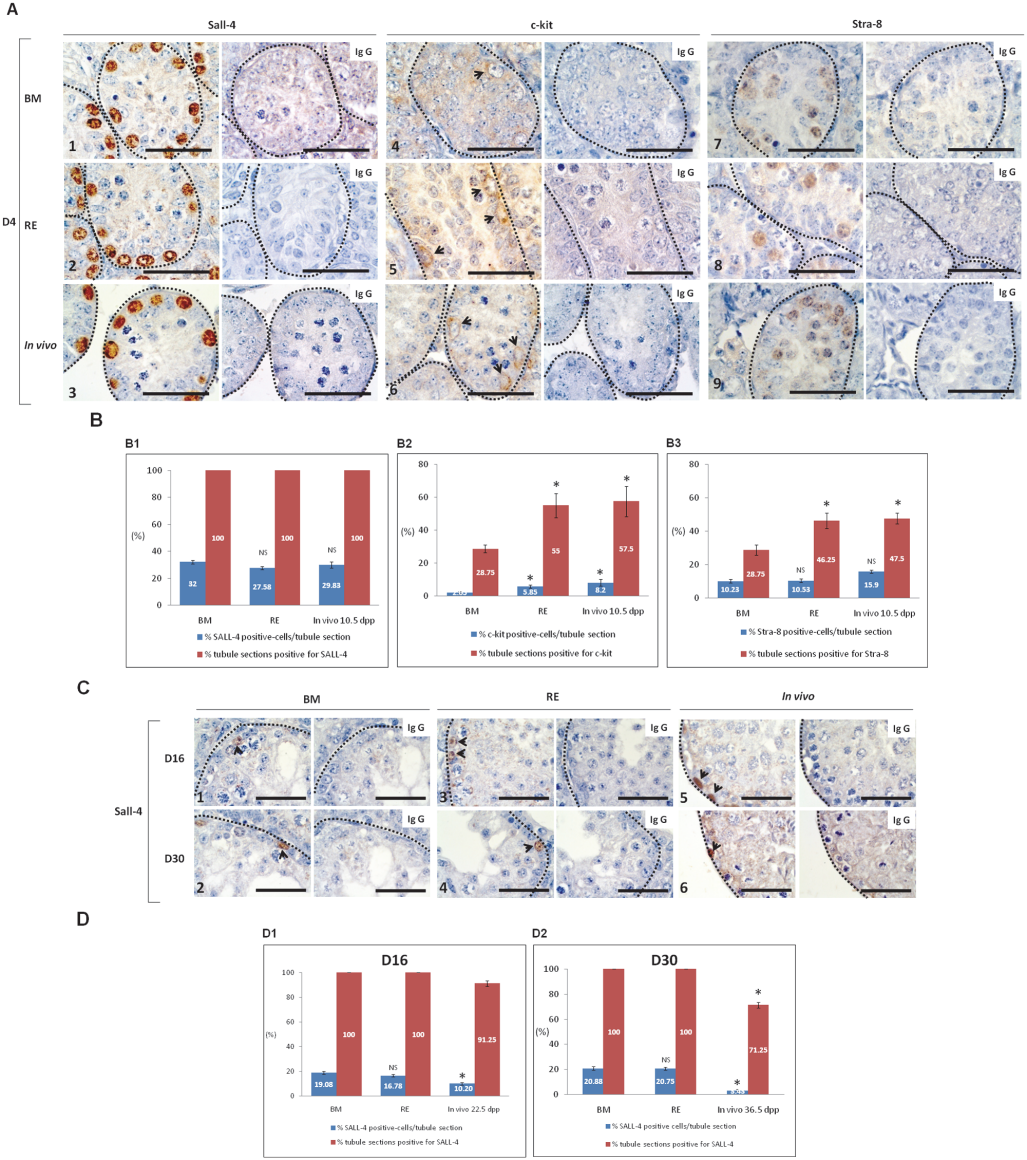
Assessment of i*n vitro* spermatogenesis initiation after immunodetection of Sall-4, c-kit and Stra-8 proteins in CD-1 prepubertal mice testes (6.5 *dpp*) at D4 and Sall-4 immunodetection at D16 and D30 of culture, in the absence or presence of RE. (**A**) Immunohistochemical detection of Sall-4, c-kit and Stra-8 on seminiferous tubule sections of prepubertal mice testes at D4 of culture in the absence (**A1–A4–A7**) or presence (**A2–A5–A8**) of RE and their corresponding *in vivo* ages (10.5 *dpp*) (**A3–A6–A9**). Brown staining was considered as a positive immunodetection of Sall-4, c-kit and Stra-8 in seminiferous tubule sections counterstained with haematoxylin. Negative control is shown on the right of each positive immunostaining. Photomicrographs were captured at a × 1000 magnification and the scale bars represent 40 μm. (**B**) Effect of RE on Sall-4, c-kit and Stra-8 expression in seminiferous tubules of prepubertal mice testes (6.5 *dpp*) cultured for 4 days *in vitro*. (**B1**) Mean proportion of Sall-4-positive cells per seminiferous tubule section (blue column) and mean proportion of Sall-4 positive tubules (red column). (**B2**) Mean proportion of c-kit positive cells per seminiferous tubule section (blue column) and mean proportion of c-kit positive tubules (red column). (**B3**) Mean proportion of Stra-8 positive cells per seminiferous tubule section (blue column) and mean proportion of Stra-8 positive tubules (red column). Values are represented as mean ± s.e.m., n = 4. Asterisk indicates a statistically significant difference between BM and RE or between BM and *in vivo* condition (*p*<0.05). (**C**) Immunohistochemical detection of Sall-4 on seminiferous tubule sections of cultured prepubertal mice testes at D16 and D30 in the absence (**C1–C2**) or presence (**C3–C4**) of RE and their corresponding *in vivo* ages (22.5 *dpp* and 36.5 *dpp*, respectively) (**C5–C6**). Brown staining was considered as a positive immunodetection of Sall-4 in seminiferous tubule sections counterstained with haematoxylin. Negative control is shown on the right of each positive immunostaining. Photomicrographs were captured at a × 1000 magnification and the scale bars represent 40 μm. (**D**) Assessment of *in vitro* maintenance of undifferentiated type A spermatogonia after immunodetection of Sall-4 at D16 and D30 of culture, in the presence or absence of RE. (**D1**) Mean proportion of Sall-4-positive cells per seminiferous tubule section (blue column) and mean proportion of Sall-4 positive tubules (red column) at D16 of culture. (**D2**) Mean proportion of Sall-4-positive cells per seminiferous tubule section (blue column) and mean proportion of Sall-4 positive tubules (red column) at D30 of culture. Values are represented as mean ± s.e.m., n = 4. Asterisk indicates a statistically significant difference between BM and RE or between BM and *in vivo* condition (*p* < 0.05). ***Footnotes*: BM**: Basal Medium, **D**: day, ***dpp***: days post partum, ***n***: Number of mice testes used in each condition, **NS**: No statistical Significance, **RE**: Retinol, **s.e.m.**: Standard Error of Mean, **IgG**: Immunoglobulin G

At D4, undifferentiated type A spermatogonia, immunostained with Sall-4, were detected in the whole cross-sectioned tubules with all tested conditions (Fig. 4A1, 4A2 and 4A3). No statistical differences were observed between BM and RE considering the proportion of Sall-4 positive spermatogonia per tubule cross section (Fig. 4B1). However, c-kit and Stra-8 immunostaining appeared stronger with RE (Fig. 4A5, 4A8), when compared with BM (Fig. 4A4, 4A7). C-kit and Stra-8 immunostaining appeared similar between RE (Fig. 4A5, 4A8) and *in vivo* (Fig. 4A6, 4A9). Moreover, RE significantly increased the proportion of c-kit positive-cells per tubule cross section in comparison to BM (p = 0.02) (Fig. 4B2), but also the proportion of c-kit (p = 0.01) and Stra-8 (p = 0.01) positive tubules when compared to BM alone (Fig. 4B2 and 4B3). RE maintains a similar expression of c-kit and Stra-8 as observed *in vivo*.

At D16 and D30, undifferentiated type A spermatogonia, immunostained with Sall-4, were detected *in vitro* with BM and RE (Fig. 4C). The whole tubule sections were positive for Sall-4 in presence or absence of RE. RE did not modify the proportion of Sall-4 positive-cells per seminiferous tubule section compared to BM (Fig. 4D1 and 4D2). However, the proportion of Sall-4 positive-cells per seminiferous tubule section was significantly increased with BM and RE conditions when compared to the *in vivo* matched-ages (p = 0.01) (Fig. 4D1 and 4D2). The proportion of Sall-4 positive-tubules was significantly increased with BM and RE when compared to *in vivo* condition at D30 (p = 0.01) (Fig. 4D2).

### RE increases spermatid and spermatozoa generation after in vitro maturation of prepubertal mice SSCs

#### Histological evaluation

**Fig 5:**
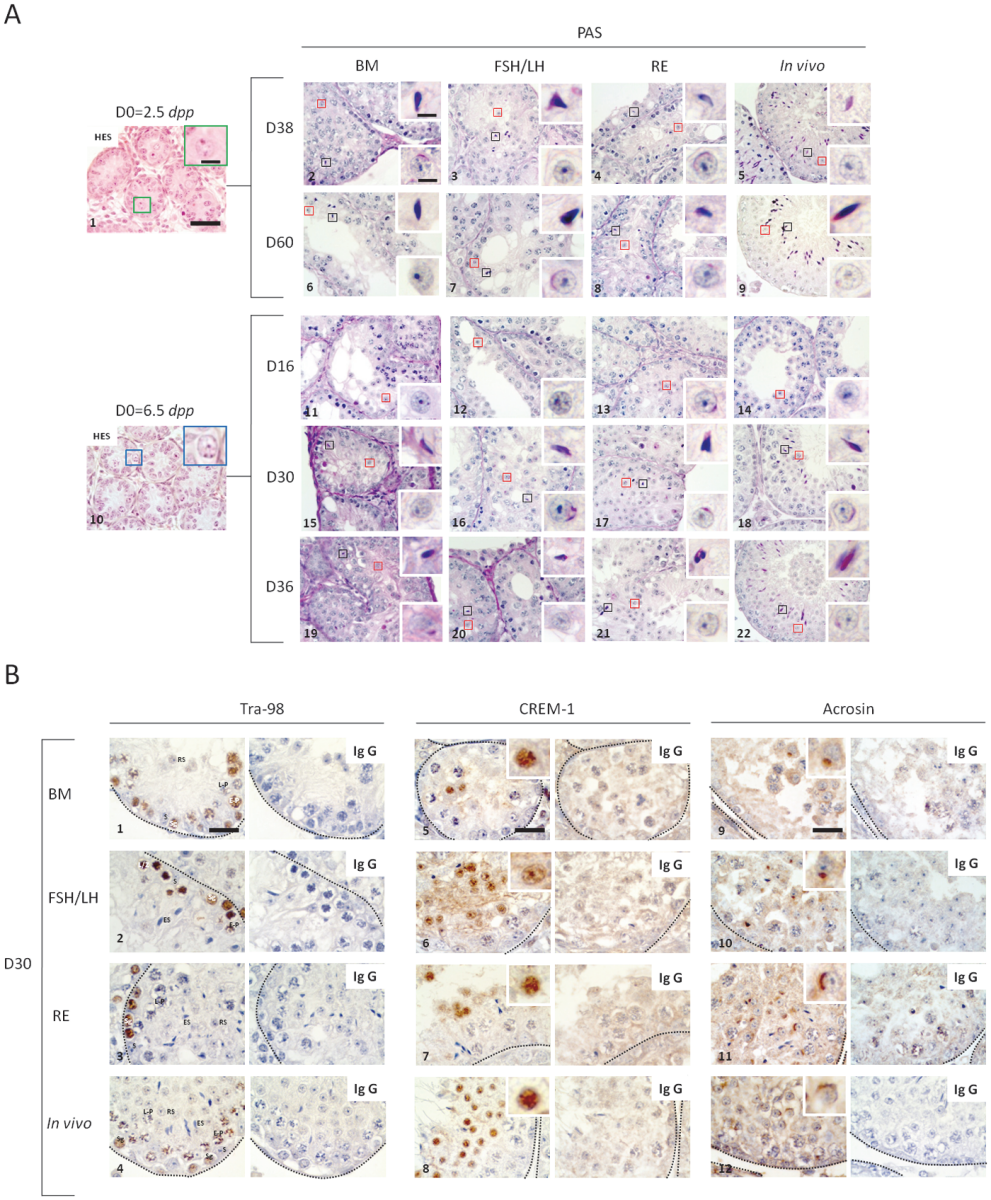
Histological evaluation of seminiferous tubules after *in vitro* maturation of prepubertal (2.5 *dpp* and 6.5 *dpp*) CD-1 mice testes. **(A)** Spermatid formation during culture from gonocytes or spermatogonia of CD-1 mice testes. Photomicrographs were captured at a ×500 (**A1** to **A22**) and ×1000 (**Insets**) magnification. The scale bar represents 40 μm or 5 μm in the photomicrograph or inset, respectively, which apply to all other photomicrographs and insets. Corresponding *in vivo* ages for the culture time points tested are also shown (**A5**–**A9**–**A14**–**A18**–**A22**). At D0 of culture, gonocytes **(A1)** (green box) or spermatogonia **(A10)** (blue box) were the only germ cells present in the seminiferous tubules. Red boxes represent round spermatids produced in each culture condition tested (BM, FSH/LH and RE) at D16, D30, D36, D38 and D60 of culture, and are enlarged in the lower inset of each photomicrograph. Black boxes represent elongated spermatids generated at each culture condition tested (BM, FSH/LH and RE) at D30, D36, D38, and D60 of culture, and are enlarged at the upper inset of each photomicrograph. (**B**) Histological evaluation of spermatogenic cell stages after Tra98, CREM-1 and Acrosin immunostaining in all conditions tested (BM, FSH/LH and RE) at D30 of culture with their corresponding *in vivo* ages (36.5 *dpp*). Photomicrographs were captured at a ×1000 magnification and the scale bars represent 20 μm. Negative controls are shown on the right of each positive immunostaining tested. (**B1–B2–B3–B4**) Brown staining indicates spermatogonia as well as leptotene/zygotene and early pachytene spermatocytes I with positive nuclear expression of Tra98. Sertoli cells, mid-pachytene and late pachytene spermatocytes I and round-to-elongated spermatids counterstain with hematoxylin only (no Tra98). **(B5–B6–B7–B8)** Immunodetection of CREM-1 as a specific marker of round spermatids that are enlarged at the inset of each photomicrograph for all culture conditions tested (BM, FSH/LH and RE). (**B9–B10–B11–B12**) Round spermatids developed a genuine acrosomal cap in all culture conditions tested (BM, FSH/LH and RE) at D30 of culture, which stains in brown using an anti-acrosin antibody, and is enlarged at the inset of each photomicrograph. ***Footnotes*: CREM:** cAMP-Responsive Element Modulator, **D**: Day, ***dpp***: day *post-partum*, **ES**: Elongated Spermatid, **E-P**: Early Pachytene Spermatocyte I, **HES**: Hemalun Eosin Saffron, **L/Z**: Leptotene/Zygotene Spermatocyte I, **L-P**: Late Pachytene Spermatocyte I, **PAS**: Periodic Acid-Schiff, **RS**: Round Spermatid, **S**: Sertoli cell, **Sg**: Spermatogonia, **IgG**: Immunoglobulin G

*(i) Cultures of 2*.*5 dpp mice testes*. At D0, seminiferous tubules contained only gonocytes and Sertoli cells (Fig. 5A1, S1A1 Fig.). Round and elongated spermatids were detected in all conditions tested at D34 (S1A2–S1A4 Fig.), D38 and D60 (Fig. 5A2–5A9). At D34, the mean percentage of elongated spermatids was significantly increased in presence of FSH/LH when compared to BM (p = 0.02). However, RE significantly increased the mean percentage of round (p = 0.01) but also elongated spermatids (p = 0.01) when compared with BM (S1A5 and S1A6 Fig.). At D38, FSH/LH significantly increased the mean proportion of round spermatids compared with BM (p = 0.02) (Table 1). However, the mean proportion of elongated spermatids was not significantly different between these two conditions at D38 or at D60 (Table 1). RE had no effect on the percentage of round spermatids observed at D38 but significantly increased the mean proportion of elongated spermatids compared with BM (p = 0.02). The mean proportion of round spermatids did not increase at D60 compared with D38 of culture whatever the condition tested (p = 0.34). The mean proportions of round and elongated spermatids are not statistically different between FSH/LH and RE at D38 and D60. Regardless of the culture medium, the proportions of round or elongated spermatids obtained were significantly lower *in vitro* at D34, D38 and D60 compared with the corresponding *in vivo* ages, i.e., 36.5 *dpp* (p = 0.01), 40.5 *dpp* (p = 0.01) and 62.5 *dpp* (p = 0.01).

**Table 1:** Assessment of germ cell differentiation using Tra-98 immunostaining of seminiferous tubules obtained after *in vitro* culture of 2.5 *dpp* testes for 38 and 60 days compared with age-matched *in vivo* tissues.

	D0 = 2.5 *dpp*							
	D38 of culture				D60 of culture			
Intra-tubular cells (%)	BM	FSH/LH	RE	*In vivo* 40.5 *dpp*	BM	FSH/LH	RE	*In vivo* 62.5 *dpp*
Sertoli cells	29.93 ± 2.12	25.00 ± 1.45	41.18 ± 1.81	7.48 ± 0.24	45.83 ± 2.41	27.65 ± 1.24	28.40 ± 2.64	5.73 ± 0.58
Spermatogonia	27.30 ± 1.51	17.88 ± 3.08	13.73 ± 0.52	8.25 ± 0.48	19.68 ± 0.74	16.80 ± 2.24	25.18 ± 1.46	8.58 ± 0.52
L/Z Spermatocytes I	13.50 ± 0.65	10.75 ± 1.71	18.58 ± 1.26	7.80 ± 0.49	14.00 ± 1.96	18.60 ± 5.81	14.53 ± 2.08	4.75 ± 0.85
P Spermatocytes I	24.85 ± 2.75	33.93 ± 0.42	20.80 ± 0.95	22.63 ± 1.79	17.40 ± 2.04	32.38 ± 1.63	26.63 ± 2.73	23.25 ± 1.75
**Round spermatids**	**4.08 ± 1.18**	11.97 ± 2.77^(*); p = 0.02^	**4.45 ± 1.01^(NS); p = 0.44^**	27.13 ± 1.24^(*); p = 0.01^	**2.45 ± 0.77**	**3.88 ± 0.72^(NS); p = 0.17^**	**4.13 ± 0.92^(NS); p = 0.1^**	29.60 ± 2.07^(*); p = 0.01^
**Elongated spermatids**	**0.35 ± 0.16**	**0.73 ± 0.13^(NS); p = 0.057^**	2.28 ± 0.31^(*); p = 0.02^	26.73 ± 0.36^(*); p = 0.01^	**0.65 ± 0.46**	**0.70 ± 0.20^(NS); p = 0.34^**	**1.15 ± 0.27^(NS); p = 0.17^**	28.10 ± 0.65^(*); p = 0.01^

The values (%) are expressed as the mean proportions ± s.e.m. of the different cell types present in the seminiferous tubules under the different culture conditions, with *n* = 4. Asterisk indicates a statistically significant difference between BM and RE or between BM and FSH/LH or between BM and *in vivo* condition concerning the percentage of round and elongated spermatids (*p*<0.05).

Footnotes:

**BM**: Basal Medium

**D**: Day

***dpp***: day *post-partum*

**FSH**: Follicle Stimulating Hormone

**LH**: Luteinizing Hormone

**L/Z**: Leptotene/Zygotene

***n***: Number of mice testes used in each condition

**NS**: Not significant

**P**: Pachytene

**s.e.m.**: Standard Error of the Mean

*(ii) Cultures of 6*.*5 dpp mice testes*. At D0, seminiferous tubules contained only Sertoli cells and spermatogonia (Fig. 5A10). At D16, the meiotic process was completed, and round spermatids were observed in all conditions tested (Fig. 5A11–5A14). Elongated spermatids were detected from D30 (Fig. 5B) to D36 in all conditions tested (Fig. 5A15–5A22 and S2 Fig.). FSH/LH significantly increased the mean percentage of round spermatids compared with BM alone at D30 of culture (p = 0.01) (Table 2). On the contrary, the mean percentage of elongated spermatids was not significantly different between these two conditions at D30 and D36. However, RE significantly increased the proportions of round (p = 0.01) and elongated spermatids (p = 0.02) when compared with BM at D30. Combining FSH/LH and RE did not lead to additive effects on the yield of round and elongated spermatids. No statistical differences were observed between FSH/LH and RE concerning the mean proportion of round and elongated spermatids at D30 and D36. Regardless of the culture medium tested, the proportions of round and elongated spermatids obtained at D30 and D36 were significantly lower than those observed at the corresponding *in vivo* ages, namely 36.5 *dpp* (p = 0.02) and 42.5 *dpp* (p = 0.01).

**Table 2:** Assessment of germ cell differentiation using Tra98 immunostaining of seminiferous tubules obtained after *in vitro* culture of 6.5 *dpp* testes for 30 and 36 days compared with age-matched *in vivo* tissues.

		D0 = 6.5 *dpp*							
		D38 of culture				D60 of culture			
Intra-tubular cells (%)	BM	FSH/LH	RE	FSH/LH + RE	*In vivo* 40.5 *dpp*	BM	FSH/LH	RE	*In vivo* 62.5 *dpp*
Sertoli cells	35.54 ± 1.58	19.24 ± 3.59	29.55 ± 2.62	27.78 ± 1.13	8.58 ± 0.44	28.92 ± 3.64	19.34 ± 2.83	30.80 ± 1.74	8.58 ± 0.58
Spermatogonia	22.93 ± 0.83	25.76 ± 5.05	21.70 ± 1.33	24.03 ± 0.88	9.68 ± 0.55	25.86 ± 1.74	24.21 ± 2.48	20.23 ± 1.69	9.18 ± 1.52
L/Z Spermatocytes I	24.98 ± 3.50	19.33 ± 5.36	18.10 ± 1.62	15.08 ± 0.67	9.28 ± 0.60	12.93 ± 1.10	14.73 ± 2.91	8.35 ± 2.10	6.90 ± 0.99
P Spermatocytes I	15.53 ± 2.75	26.33 ± 4.05	19.50 ± 1.42	29.90 ± 0.8	21.78 ± 0.48	26.75 ± 1.65	26.43 ± 0.75	28.13 ± 2.03	19.73 ± 1.36
**Round spermatids**	**0.85 ± 0.59**	7.70 ± 1.64^(*); p = 0.01^	9.10 ± 2.57^(*); p = 0.01^	**2.95 ± 2.06^(NS); p = 0.68^**	33.03 ± 0.46^(*); p = 0.01^	**4.93 ± 1.81**	**14.63 ± 6.02^(NS); p = 0.34^**	**11.45 ± 1.38^(NS); p = 0.11^**	29.25 ± 0.70^(*); p = 0.01^
**Elongated spermatids**	**0.18 ± 0.10**	**1.65 ± 1.05^(NS); p = 0.68^**	2.05 ± 0.65^(*); p = 0.02^	**0.28 ± 0.14^(NS); p = 0.68^**	**17.68 ± 0.63^(NS); p = 0.01^**	**0.63 ± 0.29**	**0.68 ± 0.42^(NS); p = 0.88^**	**1.05 ± 0.13^(NS); p = 0.2^**	26.38 ± 1.43^(*); p = 0.1^

The values (%) are expressed as the mean proportions ± s.e.m. of the different cell types present in the seminiferous tubules under the different culture conditions, with *n* = 4. Asterisk indicates a statistically significant difference between BM and RE or between BM and FSH/LH or between BM and *in vivo* condition concerning the percentage of round and elongated spermatids (*p*<0.05).

Footnotes:

**BM**: Basal Medium

**D**: Day

***dpp***: day *post-partum*

**FSH**: Follicle Stimulating Hormone

**LH**: Luteinizing Hormone

**L/Z**: Leptotene/Zygotene

***n***: Number of mice testes used in each condition

**NS**: Not significant

**P**: Pachytene

**s.e.m.**: Standard Error of the Mean

*(iii) Culture of 2*.*5 dpp and 6*.*5 dpp old testes during one wave of in vitro spermatogenesis*. The mean percentage of round spermatids were not significantly different between D34 (2.5 *dpp* old testes) and D30 (6.5 *dpp* old testes) with BM or RE (S1A5 Fig.). However, with FSH/LH, the mean percentage of round spermatids observed was significantly higher at D30 when compared with D34 (p = 0.02) (S1A5 Fig.). The mean percentage of elongated spermatids did not significantly vary between D30 and D34 regardless of the condition tested (S1A6 Fig.).

#### Sperm enumeration

*(i) Cultures of 2*.*5 dpp mice testes*. Spermatozoa were observed at D34 using Shorr staining (S1B1 Fig.). FSH/LH did not significantly increase the mean number of spermatozoa when compared with BM (S1B2 Fig.). However, the mean number of spermatozoa was significantly increased with RE when compared with BM at D34 (p = 0.02) (S1B2 Fig.).

**Fig 6:**
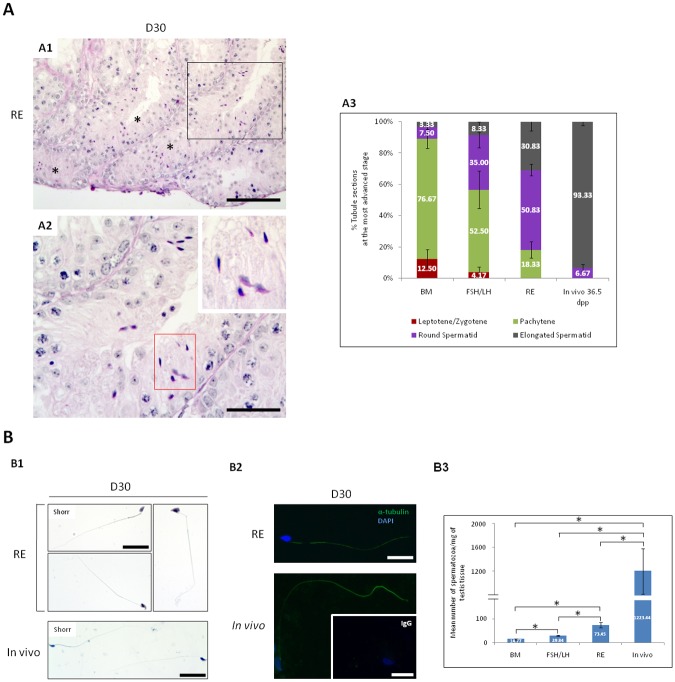
Detection and enumeration of flagellated sperm after the dissection of testes explants (at 6.5 *dpp*) cultured for 30 days. (**A**) Seminiferous tubules with spermatids as the most advanced stage at D30 of culture. Photomicrographs were captured at a ×200 (**A1**) and ×500 (**A2**) magnifications and the scale bars represent 100 μm or 40 μm, respectively. (**A1**) Multiple elongated spermatids obtained in different seminiferous tubules (black asterisks) in the presence of RE, are shown in thin sections after PAS and haematoxylin staining. Black box in **A1** is enlarged in **A2**. Red box that shows elongated spermatids is enlarged in the inset of the photomicrograph. (**A3**) Mean proportion of seminiferous tubules containing differentiated germ cells as the most advanced stage under all conditions tested at D30 of culture. (**B**) Detection and enumeration of flagellated sperm under all conditions tested at D30 of culture. Photomicrographs were captured at a ×500 (Shorr staining) and ×600 (α-tubulin detection) magnifications and the scale bars represent 40 μm. (**B1**) Flagellated sperm derived *in vitro* at D30 of culture in the presence of RE with the *in vivo*-matched age (36.5 *dpp*), as a control sperm are shown using Shorr staining. (**B2**) Genuine sperm flagella are shown in green using a specific anti-acetylated α-tubulin antibody, and nuclei are stained in blue by DAPI. A negative control was performed with a pre-immune mouse IgG and is shown in the inset of the photomicrograph. (**B3**) Mean number of spermatozoa produced in 6.5 *dpp* testicular tissues cultured for 30 days under the conditions tested in the present study.

*(ii) Cultures of 6*.*5 dpp mice testes*. The highest rates of elongated spermatids were obtained with RE at D30 (Table 2). Therefore, 6.5 day-old testes were cultured for sperm enumeration during 30 days of culture with BM, FSH/LH or RE. Multiple elongated spermatids were obtained in several seminiferous tubules with RE (Fig. 6A1 and 6A2). Compared with BM, RE increased the mean proportion of seminiferous tubules containing elongated spermatids as the most advanced stage (p = 0.02) (Fig. 6A3). Spermatozoa obtained at D30 were confirmed by Shorr staining (Fig. 6B1), and genuine flagella were detected by immunofluorescence with an anti-acetylated tubulin antibody (Fig. 6B2). FSH/LH significantly increased the mean number of flagellated spermatozoa compared with BM (p = 0.02). However, more numerous spermatozoa were obtained with RE compared with BM or FSH/LH (p = 0.02) (Fig. 6B3). Regardless of the culture medium tested, the mean number of flagellated spermatozoa produced *in vitro* was significantly lower than that observed at the corresponding *in vivo* age, namely 36.5 *dpp* (p = 0.01) (Fig. 6B3).

Results are represented as the mean ± s.e.m., *n* = 4. Asterisk indicates a statistically significant difference between BM and FSH/LH or between BM and RE or between FSH/LH and RE (*p* = 0.02) or between *in vitro* and *in vivo* conditions (*p* = 0.01). ***Footnotes*: BM**: Basal Medium, **DAPI**: 4’, 6’-diamino-2-phenylindol, ***dpp***: day *post-partum*, **FSH**: Follicle Stimulating Hormone, **LH**: Luteinizing Hormone, **mg**: milligram, ***n***: Number of mice testes used in each condition, **PAS**: Periodic Acid-Schiff, **s.e.m.**: Standard Error of the Mean**, IgG**: Immunoglobulin G

*(iii) Cultures of 2*.*5 dpp and 6*.*5 dpp old testes during one wave of in vitro spermatogenesis*. The mean number of spermatozoa were not significantly different between D34 (2.5 *dpp* old testes) and D30 (6.5 *dpp* old testes) with BM or FSH/LH (S1B2 Fig.). However, the mean number of spermatozoa was significantly higher at D30 when compared to D34 with RE (p = 0.01).

### RE maintains in vitro Leydig cell functional integrity, while FSH/LH increases testosterone concentration during in vitro culture of pre-pubertal mice testes (6.5 dpp)

Testosterone production increased significantly from D0 to D36 with BM (p = 0.0071), FSH/LH (p = 0.0009) or RE (p = 0.0062), reflecting the well-preserved functionality of Leydig cells *in vitro* (S3 Fig.). However, a significant increase of testosterone concentration was obtained with FSH/LH compared to BM at D11, D18, D21, D30 and D36 (p = 0.02) and compared to RE at D11, D30 and D36 (p = 0.02). The testosterone levels were similar between RE and BM at each culture time point tested (p>0.05) (S3 Fig.).

## Discussion

In this study, the integrity and structure (i.e., structural modifications) of the seminiferous epithelium were assessed, and Leydig cell functional integrity was evaluated based on the steroidogenic activity observed at different times in organotypic culture of pre-pubertal mice testes. We showed that RE significantly enhanced the *in vitro* production of spermatids and spermatozoa from mice SSCs at D30 of culture.

Our results reported that spermatozoa can be generated from SSCs from both 2.5 and 6.5 *dpp* mouse pups using an organ culture system. Indeed, round and elongated spermatids were detected at D16 and D30, respectively, after *in vitro* culture of 6.5 *dpp* mice testes, as proposed by Sato *et al*. [11]. Therefore, our results are consistent with the data reported by Gohbara *et al*. [12] (2010) showing *in vitro Gsg2*-GFP expression (indicating the end stages of meiosis) around the age corresponding to 19–25 *dpp*. Furthermore, our study suggested that the first wave of spermatogenesis occurred *in vitro* in a physiologically time-dependent manner. Round spermatids were histologically detectable at D16 *in vitro* and between D21 and D23 *in vivo* in CD-1 mice testes; elongated spermatids and flagellated spermatozoa were observed at D30 *in vitro* and between D30 and D36 *in vivo*.

Thereafter, we attempted to improve the *in vitro* spermatogenic differentiation of gonocytes or SSCs by supplementing the basal medium with RE, alone FSH/LH alone or a combination of both. In this study, the RE concentration was based on the study of Travers *et al*. [18] demonstrating that RE at a concentration of 10^-6^ M, rather than RA, was able to maintain germ cell proliferation and their ability to initiate spermatogenesis [18]. During the first wave of spermatogenesis, the retinaldehyde dehydrogenase (RALDH)-dependent synthesis of RA by Sertoli cells is absolutely necessary to initiate the differentiation of A-aligned into A1 spermatogonia [16]. In the current study, we attempted to mimic the physiological *in vivo* conditions of the delivery of RA to germ cells, which occurs in a temporally and spatially controlled manner.

The present study showed that RE significantly enhanced the *in vitro* production of spermatids and spermatozoa from neonatal (2.5 *dpp* and 6.5 *dpp*) mice testes. It was previously demonstrated that RE stimulates the DNA replication of gonocytes but also their differentiation in cultured neonatal mice testes [17]. In addition, our data are supported by previous findings in VAD prepubertal [23] or adult mice [24, 25], in which spermatogenesis is impaired, leading to the blockage of spermatogonia differentiation and the accumulation of undifferentiated spermatogonia in the seminiferous tubules. After vitamin A replacement in adult mice, spermatogenesis is reinitiated and the seminiferous epithelium becomes stage-synchronized, leading to the formation of spermatocytes and spermatids. These data suggest that vitamin A is required for the full development of spermatogenic cells into spermatozoa [24, 25]. *In vitro*, RE also promotes the self-renewal of male germline stem cells (mGSCs) and the maintenance of mGSC pluripotency in adult mice [26]. More recently, Gohbara *et al*. [12] demonstrated that the disruption of RA action by a retinoic acid receptor pan-antagonist (LE540) prevented spermatogenesis *in vitro* as it does *in vivo via* the partial inhibition of meiosis and the consequent decrease in round spermatid production. The male germ cells do not enter meiosis until 8–10 *dpp*, as evidenced by the first appearance of preleptotene spermatocytes. Therefore, in our study, we chose to stop cultures of 6.5 day-old mice testes at D4 of culture to explore specifically the onset of meiosis. Although RE was added to the culture medium, the mean proportion of Sall-4 positive-cells per tubule cross section was not modified in comparison with BM, at D4, D16 and D30. In addition, the whole tubules were positive for Sall-4 under all tested conditions during *in vitro* culture. Therefore, the pool of undifferentiated spermatogonia was maintained at the beginning of spermatogenesis but also at the later time points in this organ culture system. However, RE increased the proportion of differentiating c-kit positive spermatogonia per tubule section, but also the proportion of tubule sections containing differentiating c-kit positive spermatogonia and Stra-8 positive preleptotene spermatocytes at the onset of meiosis (D4) (Fig. 4B2 and 4B3). This is the first evidence that RE promoted spermatogenesis initiation and germ cell differentiation from pre-pubertal mice testes in our organ culture system. Therefore, our findings suggest that RE i) does not prevent self-renewal of SSCs, ii) enhances SSC differentiation and their entry into meiosis in a more elevated proportion of seminiferous tubules and iii) promotes haploid germ cell production *in vitro*. Indeed, our results showed an increased proportion of seminiferous tubules containing round or elongated spermatids as the most advanced stage when RE was added to the basal medium containing only KSR. Therefore, we chose to dissect testes explants at this time point (D30), where the best rates of spermatids were obtained, to identify and count spermatozoa that were never observed on histological sections. Indeed, spermatozoa were detected after dissection of cultured testes explants in all conditions tested, but the highest number of spermatozoa were observed with RE. This finding is in agreement with our results obtained with histological analyses showing the highest rates of elongated spermatids per seminiferous tubules. Several studies previously described the molecular mechanism through which RE acts on the onset of meiosis [16]. However, to the best of our knowledge, there is no study that describes the molecular mechanism by which RE enhances the differentiation of round spermatids into spermatozoa, although the presence of specific receptors of retinoic acid (RXR or RAR) in round spermatids were previously reported [27]. In addition, we noticed that RE influenced positively other objective parameters than the number of generated sperm; notably seminiferous epithelium integrity that was better maintained compared to BM or FSH/LH. Therefore, the molecular mechanism and intracellular signalling pathways through which RE acts on spermiogenesis should be identified to better understand and further improve this process *in vitro*.

In parallel, we demonstrated that, compared with the basal medium, FSH/LH alone increased round spermatid formation *in vitro* at D30 and D38. The role of these hormones in the regulation of spermatogenesis has been clarified through studies of mice lacking specific hormone receptors. Indeed, in FSHRKO.SCARKO mice, germ cells enter meiosis; however, development arrests at early pachytene for most cells, and no secondary spermatocytes or apparent round spermatids are produced [28]. Consequently, FSH and androgen appear to have an additive effect on the entry into meiosis but act synergistically to stimulate the completion of meiosis and entry into spermiogenesis [28]. Moreover, no additive effect was observed when FSH/LH and RE were combined in the medium. Notably, interactions between retinoids and FSH, the main hormonal regulator of Sertoli cell function, are complex. In mouse Sertoli cell lines, RA inhibits FSH transduction pathways by blocking the production of cAMP; in return, FSH reduces the expression of RAR-α, which is one of the major RA receptors [29, 30]. Therefore, these data could support our observation that the simultaneous presence of RE and FSH in the culture medium could negatively influence the course of germ cell differentiation through spermatogenesis steps. Thus, FSH and RE are likely to act in a dose- and time-dependent manner *in vivo* that unfortunately is difficult to reproduce specifically *in vitro*. Furthermore, 2.5 *dpp* and 6.5 *dpp* old testes cultured for 34 and 30 days did not proceed similarly in term of the percentage of round spermatids generated in presence of FSH/LH but also in term of the mean number of flagellated spermatozoa generated in the presence of RE. Indeed, RE enhanced sperm production at D30 (6.5 *dpp* old testes) when compared to D34 (2.5 *dpp* old testes). This finding could be due to the fact that gonocytes have already achieved mitosis and their migration to the basement membrane giving rise to type A spermatogonia in 6.5 *dpp* old testes [31]. Then, germ cells are in a more advanced stage when culture starts from 6.5 *dpp* old testes which could influence positively *in vitro* germ cell differentiation. Therefore, we can suggest that the efficiency of *in vitro* spermatogenesis depends on the culture medium composition but also on the germ stem cell stage present at the beginning of the culture. Moreover, our organ culture conditions did not sufficiently mimic the *in vivo* condition, and the normal progress of spermatogenesis could not be reproduced for all the culture periods tested. Hence, further investigations should be made in the future to better improve the efficiency of *in vitro* spermatogenesis. We also noticed in this study that the lowest rates of spermatids produced *in vitro* from 2.5 *dpp* mice testes were observed at the latest time point corresponding to D60, regardless of the culture conditions tested. This is in agreement with the study of Sato *et al*. [12] that reported a decrease in *Gsg2*-GFP (a marker of meiosis end) expression from D45 to D60 when cultures were started from 2.5 days old testes, and suggested that this organ culture system was able to maintain *in vitro* spermatogenesis for a long time period.

To the best of our knowledge, seminiferous tubule growth and intratubular cell proliferation have not yet been assessed in the organ culture system described here. Therefore, our results provide the first evidence of seminiferous tubule growth in culture and the maintenance of intra-tubular cell proliferation with no deleterious effects in the presence of RE. During the first wave of spermatogenesis, FSH and androgens have additive effects on the maintenance of germ cell viability. An increased germ cell loss was observed in FSHRKO.ARKO mice, leading to a dramatic decrease in the germ/Sertoli cell ratio, tubule diameter and testis volume [32]. In our study, the growth of seminiferous tubules was positively and significantly correlated with the germ/Sertoli cell ratio for all the culture conditions using 6.5 d*pp* old testis. However, seminiferous tubule growth was more pronounced when FSH and LH were added to the basal medium, which was likely due to the higher germ/Sertoli cell ratio [32].

Testosterone production increased over the course of the culture under all conditions tested, consistent with the preservation of the functional integrity of Leydig cells. Furthermore, testosterone levels increased between D11 and D36 when LH was added to the medium beginning at D7 of culture in agreement with the physiological *in vivo* establishment of the normal adult cohort of Leydig cells [33]. In contrast, no significant increase in testosterone levels was induced by RE, although greater proportions of spermatids and spermatozoa were detected in this condition. Our data also supported the observation that RE and RA had no effect on basal or LH-stimulated testosterone production in neonatal or adult testes [34]. Therefore, it can also be suggested that a low level of testosterone is required during the first wave of spermatogenesis. On the contrary, the greater LH-stimulated testosterone production between D11 and D36 reported in the FSH-LH condition may have a detrimental effect on spermatogonial proliferation, meiosis and seminiferous epithelium integrity [35].

In conclusion, both RE and FSH/LH influenced the course of *in vitro* spermatogenesis. However, we demonstrated that RE improved the *in vitro* production of spermatids and spermatozoa after the culture of prepubertal mouse SSCs at a gas-liquid interphase. RE may allow the generation of a sufficient pool of differentiating germ cells able to undergo complete *in vitro* differentiation. Furthermore, RE treatment maintained the integrity of the seminiferous epithelium as well as tubular growth and intra-tubular cell proliferation. These organ culture conditions could be a useful tool for *in vitro* spermatogenesis from pre-pubertal human testicular tissue.

## Supporting Information

S1 Fig Histological evaluation and sperm enumeration in prepubertal (2.5 *dpp* and 6.5 *dpp*) mice testes cultured during one wave of spermatogenesis. (**A**) Spermatid formation during a period of 34 days of culture from gonocytes of CD-1 mice testes. At D0 of culture, gonocytes **(A1)** (white box) were the only germ cells present in the seminiferous tubules. Green boxes represent round spermatids produced in each culture condition tested (BM, FSH/LH and RE) at D34 of culture, and are enlarged in the lower inset of each photomicrograph. Blue boxes represent elongated spermatids generated in each culture condition tested (BM, FSH/LH and RE) at D34 of culture, and are enlarged at the upper inset of each photomicrograph. Photomicrographs were captured at ×500 (**A1** to **A4**) and ×1000 (**Insets**) magnification. The scale bar represents 40 μm or 5 μm in the photomicrograph or inset, respectively, which apply to all other photomicrographs and insets. The proportion of round (**A5**) and elongated (**A6**) spermatids per seminiferous tubule were obtained from 2.5 *dpp* and 6.5 *dpp* old testes after one wave of *in vitro* spermatogenesis. The values (%) are expressed as the mean proportions ± s.e.m. of round and elongated spermatids present in the seminiferous tubules under the different culture conditions, with *n* = 4. Note that culture conditions tested for *in vitro* culture of 2.5 *dpp* old testis were compared and (a) symbol indicates a statistically significant difference between BM and RE or between BM and FSH/LH concerning the percentage of round and elongated spermatids (*p*<0.05). Asterisk indicates a statistically significant difference between 2.5 *dpp* and 6.5 *dpp* in term of spermatid proportion obtained in each culture condition tested (*p* = 0.02). (**B**) Sperm enumeration in prepubertal (2.5 *dpp* and 6.5 *dpp*) mice testes cultured during one wave of *in vitro* spermatogenesis. (**B1**) Flagellated spermatozoa generated in presence of RE were detected after the dissection of 2.5 *dpp* old testes cultured during 34 days. Photomicrographs were captured at ×500 (Shorr staining) magnification and the scale bar represents 40 μm. (**B2**) Mean number of spermatozoa produced in 2.5 *dpp* and 6.5 *dpp* testicular tissues cultured during one wave of *in vitro* spermatogenesis under the conditions tested. Results are represented as the mean ± s.e.m., *n* = 4. Note that culture conditions tested for *in vitro* culture of 2.5 *dpp* old testis were compared and (a) symbol indicates a statistically significant difference between BM and RE or between BM and FSH/LH concerning the mean number of spermatozoa produced per milligram of testis tissue (*p* = 0.02). Asterisk indicates a statistically significant difference between 2.5 *dpp* and 6.5 *dpp* in term of the mean number of spermatozoa generated per milligram of testis tissue (*p* = 0.01). ***Footnotes*: BM**: Basal Medium, **D**: Day, ***dpp***: day *post partum*, **FSH**: Follicle Stimulating Hormone, **LH**: Luteinizing Hormone, **mg**: milligram, ***n***: Number of experiments for each condition, **NS**: No significance, **PAS**: Periodic Acid-Schiff, **RE**: Retinol, **s.e.m.:** Standard Error of the Mean
(TIF)

S2 Fig Histological evaluation of seminiferous tubules in the presence of FSH/LH and RE simultaneously in the culture medium after *in vitro* maturation of 6.5 *dpp* mice testes during 30 days. (**A**) At D0 of culture, spermatogonia (blue box) were the only germ cells present in the seminiferous tubules and are enlarged in the upper inset of the photomicrograph. (**B**) At D30 of culture, round (red box) and elongated (black box) spermatids were detected when FSH/LH and RE were present in the culture medium. (**C**) Round and elongated spermatids of the age-matched *in vivo* (36.5 *dpp*) are shown with red and black boxes, respectively. Round and elongated spermatids are enlarged in the lower and upper inset of each photomicrograph, respectively. Photomicrographs were captured at ×500 (**A** to **C**) and ×1000 (**Insets**) magnification. The scale bar represents 40 μm or 5 μm in the photomicrograph or inset, respectively, which apply to all other photomicrographs and insets. ***Footnotes***: **D**: Day, ***dpp***: day *post-partum*, **FSH**: Follicle Stimulating Hormone, **HES**: Hemalun Eosin Saffron, **LH**: Luteinizing Hormone, **PAS**: Periodic Acid-Schiff, **RE**: Retinol
(TIF)

S3 Fig *In vitro* production of testosterone from testes explants (at 6.5 *dpp*) cultured for a 36 day period. The results are represented as the mean ± s.e.m., *n* = 4. Asterisk indicates a statistically significant difference between BM and FSH/LH condition (*p*<0.05). ***Footnotes*: BM**: Basal Medium, **D**: Day, **FSH**: Follicle Stimulating Hormone, **LH**: Luteinizing Hormone, ***n***: Number of experiments for each condition, **RE**: Retinol, **s.e.m.:** Standard Error of the Mean
(TIF)

S1 Table Assessment of germ cell differentiation using Tra98 immunostaining of seminiferous tubules obtained after *in vitro* culture of 6.5 *dpp* testes for 30 days with different concentrations of FSH, LH alone or in combination supplemented to the BM from D0 of culture. The values (%) are expressed as the mean proportions of the different cell types present in the seminiferous tubules under the different culture conditions, with *n* = 1. ***Footnotes*: BM**: Basal Medium, **D**: Day, ***dpp***: day *post-partum*, **FSH**: Follicle Stimulating Hormone, **LH**: Luteinizing Hormone, **L/Z**: Leptotene/Zygotene, ***n***: Number of mice testes used in each condition, **P**: Pachytene
(XLSX)

S2 Table Assessment of germ cell differentiation using Tra-98 immunostaining of seminiferous tubules obtained after *in vitro* culture of 6.5 *dpp* testes for 30 days with different concentrations of FSH, LH alone or in combination supplemented to the BM from D7 of culture. The values (%) are expressed as the mean proportions of the different cell types present in the seminiferous tubules under the different culture conditions, with *n* = 1. ***Footnotes*: BM**: Basal Medium, **D**: Day, ***dpp***: day *post-partum*, **FSH**: Follicle Stimulating Hormone, **LH**: Luteinizing Hormone, **L/Z**: Leptotene/Zygotene, ***n***: Number of mice testes used in each condition, **P**: Pachytene
(XLSX)

S3 Table Assessment of germ cell differentiation using Tra-98 immunostaining of seminiferous tubules obtained after *in vitro* culture of 6.5 *dpp* testes for 30 days with different concentrations of FSH, LH alone or in combination supplemented to the BM from D14 of culture. The values (%) are expressed as the mean proportions of the different cell types present in the seminiferous tubules under the different culture conditions, with *n* = 1. ***Footnotes*: BM**: Basal Medium, **D**: Day, ***dpp***: day *post-partum*, **FSH**: Follicle Stimulating Hormone, **LH**: Luteinizing Hormone, **L/Z**: Leptotene/Zygotene, ***n***: Number of mice testes used in each condition, **P**: Pachytene
(XLSX)
