# Correction: Retinol Improves *In Vitro* Differentiation of Pre-Pubertal Mouse Spermatogonial Stem Cells into Sperm during the First Wave of Spermatogenesis

**DOI:** 10.1371/journal.pone.0123846

**Published:** 2015-04-02

**Authors:** The PLOS ONE Staff

There are errors in Table 1 and Table 2 of the published article. Please view the correct Table 1 and Table 2 here.

**Table 1:** Assessment of germ cell differentiation using Tra-98 immunostaining of seminiferous tubules obtained after *in vitro* culture of 2.5 *dpp* testes for 38 and 60 days compared with age-matched *in vivo* tissues. The values (%) are expressed as the mean proportions ± s.e.m. of the different cell types present in the seminiferous tubules under the different culture conditions, with *n* = 4. Asterisk indicates a statistically significant difference between BM and RE or between BM and FSH/LH or between BM and *in vivo* condition concerning the percentage of round and elongated spermatids (*p*<0.05).

	D0 = 2.5 *dpp*							
	D38 of culture				D60 of culture			
Intra-tubular cells (%)	BM	FSH/LH	RE	*In vivo* 40.5 *dpp*	BM	FSH/LH	RE	*In vivo* 62.5 *dpp*
Sertoli cells	29.93±2.12	25.00±1.45	41.18±1.81	7.48±0.24	45.83±1.24	27.65±1.24	28.40±2.64	5.73±0.58
Spermatogonia	27.30±1.51	17.88±3.08	13.73±0.52	8.28±0.48	19.68±0.74	16.80±2.24	25.18±1.46	8.58±0.52
L/Z Spermatocytes I	13.50±0.65	10.50±1.71	18.58±1.26	7.80±0.49	14.00±1.96	18.60±5.81	14.53±2.08	4.75±0.85
P Spermatocytes I	24.85±2.75	33.93±0.42	20.80±0.95	22.63±1.79	17.40±2.04	32.38±1.63	26.63±2.73	23.25±1.75
**Round spermatids**	**4.08±1.18**	11.98±2.77^(*); p = 0.02^	**4.45±1.01^(NS); p = 0.44^**	27.13±1.24^(*); p = 0.01^	**2.45±0.77**	**3.88±0.72^(NS); p = 0.17^**	**4.13±0.92^(NS); p = 0.1^**	29.60±2.07^(*); p = 0.01^
**Elongated spermatids**	**0.35±0.16**	**0.73±0.13^(NS); p = 0.057^**	1.28±0.31^(*); p = 0.02^	26.73±0.36^(*); p = 0.01^	**0.65±0.46**	**0.70±0.20^(NS); p = 0.34^**	**1.15±0.27^(NS); p = 0.17^**	28.10±0.65^(*); p = 0.01^

***Footnotes***:

**BM**: Basal Medium

***dpp***: day *post-partum*

**LH**: Luteinizing Hormone

***n***: Number of mice testes used in each condition

**P**: Pachytene

**D**: Day

**FSH**: Follicle Stimulating Hormone

**L/Z**: Leptotene/Zygotene

**NS**: Not significant

**s.e.m.**: Standard Error of the Mean

**Table 2:** Assessment of germ cell differentiation using Tra98 immunostaining of seminiferous tubules obtained after *in vitro* culture of 6.5 *dpp* testes for 30 and 36 days compared with age-matched *in vivo* tissues. The values (%) are expressed as the mean proportions ± s.e.m. of the different cell types present in the seminiferous tubules under the different culture conditions, with *n* = 4. Asterisk indicates a statistically significant difference between BM and RE or between BM and FSH/LH or between BM and *in vivo* condition concerning the percentage of round and elongated spermatids (*p*<0.05).

	D0 = 6.5 *dpp*								
	D30 of culture					D36 of culture			
Intra-tubular cells (%)	BM	FSH/LH	RE	FSH/LH + RE	*In vivo* 36.5 *dpp*	BM	FSH/LH	RE	*In vivo* 42.5 *dpp*
Sertoli cells	35,54±1.58	19.24±3.59	29.55±2.62	27.78±1.13	8.58±0.44	28.92±3.64	19.34±2.83	30.80±1.74	8.58±0.58
Spermatogonia	22.93±0.83	25.76±5.05	21.70±1.33	24.03±0.88	9.68±0.55	25.86±1.74	24.21±2.48	20.23±1.69	9.18±1.52
L/Z Spermatocytes I	24.98±3.50	19.33±5.36	18.10±1.62	15.08±0.67	9.28±0.60	12.93±1.10	14.73±2.91	8.35±2.10	6.90±0.99
P Spermatocytes I	15.53±2.75	26.33±4.05	19.50±1.42	29.90±0.08	21.78±0.48	26.75±1.65	26.43±0.75	28.13±2.03	19.73±1.36
**Round spermatids**	**0.85±0.59**	7.70±1.64^(*); p = 0.01^	9.10±2.57^(*); p = 0.01^	**2.95±2.06^(NS); p = 0.68^**	33.03±0.46^(*); p = 0.01^	**4.93±1.81**	**14.63±6.02^(NS); p = 0.34^**	**11.45±1.38^(NS); p = 0.11^**	29.25±0.70^(*); p = 0.01^
**Elongated spermatids**	**0.18±0.10**	**1.65±1.05^(NS); p = 0.68^**	2.05±0.65^(*); p = 0.02^	**0.28±0.14^(NS); p = 0.68^**	17.68±0.53^(*); p = 0.01^	**0.63±0.29**	**0.68±0.42^(NS); p = 0.88^**	**1.05±0.13^(NS); p = 0.02^**	26.38±1.43^(*); p = 0.01^

***Footnotes***:

**BM**: Basal Medium

***dpp***: day *post-partum*

**LH**: Luteinizing Hormone

***n***: Number of mice testes used in each condition

**P**: Pachytene

**D**: Day

**FSH**: Follicle Stimulating Hormone

**L/Z**: Leptotene/Zygotene

**NS**: Not significant

**s.e.m.**: Standard Error of the Mean
