# Supplementary figures and images for: Retinol Improves In Vitro Differentiation of Pre-Pubertal Mouse Spermatogonial Stem Cells into Sperm during the First Wave of Spermatogenesis
Source: PLoS One. 2015 Feb 25;10(2):e0116660. doi: 10.1371/journal.pone.0116660 (PMC4340963; doi:10.1371/journal.pone.0116660)

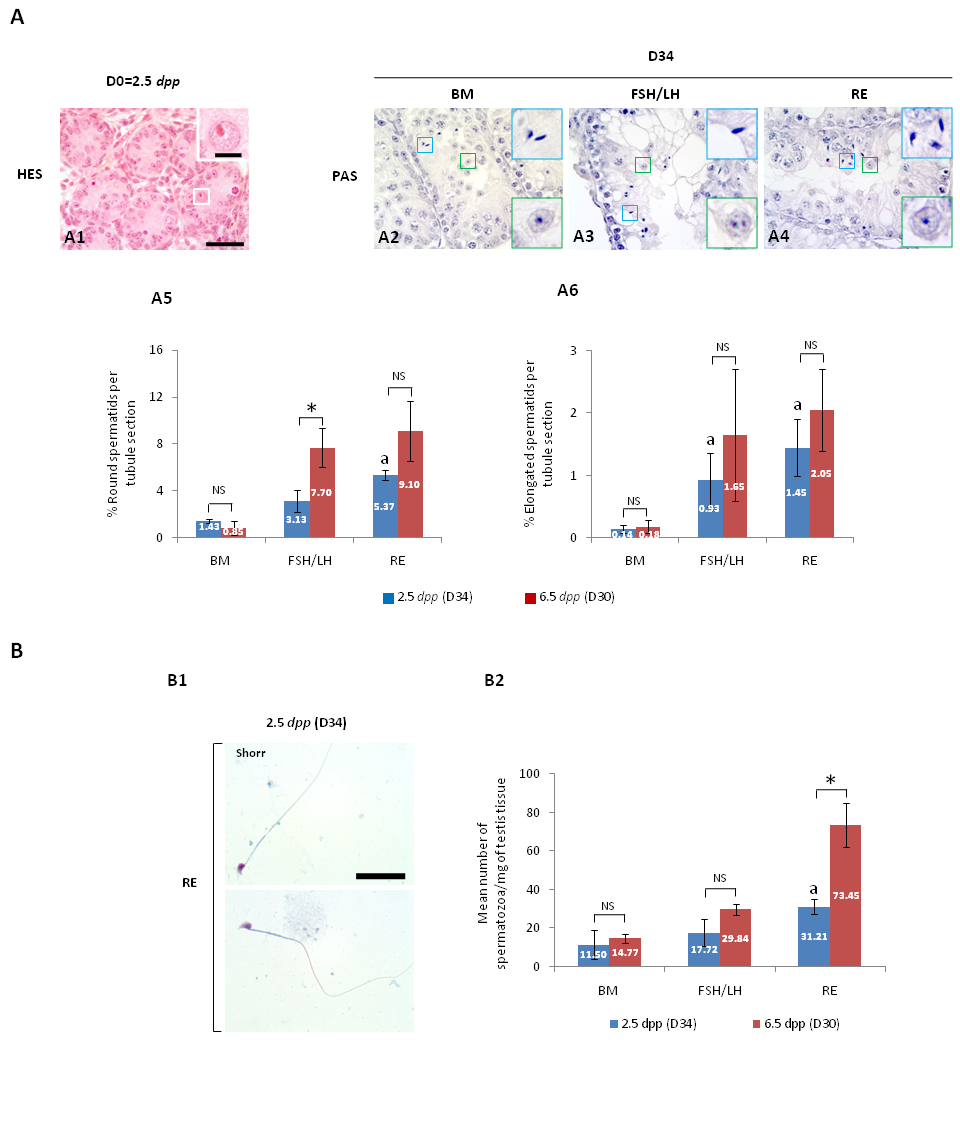

Supplement: S1 Fig — (A) Spermatid formation during a period of 34 days of culture from gonocytes of CD-1 mice testes. At D0 of culture, gonocytes (A1) (white box) were the only germ cells present in the seminiferous tubules. Green boxes represent round spermatids produced in each culture condition tested (BM, FSH/LH and RE) at D34 of culture, and are enlarged in the lower inset of each photomicrograph. Blue boxes represent elongated spermatids generated in each culture condition tested (BM, FSH/LH and RE) at D34 of culture, and are enlarged at the upper inset of each photomicrograph. Photomicrographs were captured at ×500 (A1 to A4) and ×1000 (Insets) magnification. The scale bar represents 40 μm or 5 μm in the photomicrograph or inset, respectively, which apply to all other photomicrographs and insets. The proportion of round (A5) and elongated (A6) spermatids per seminiferous tubule were obtained from 2.5 dpp and 6.5 dpp old testes after one wave of in vitro spermatogenesis. The values (%) are expressed as the mean proportions ± s.e.m. of round and elongated spermatids present in the seminiferous tubules under the different culture conditions, with n = 4. Note that culture conditions tested for in vitro culture of 2.5 dpp old testis were compared and (a) symbol indicates a statistically significant difference between BM and RE or between BM and FSH/LH concerning the percentage of round and elongated spermatids (p<0.05). Asterisk indicates a statistically significant difference between 2.5 dpp and 6.5 dpp in term of spermatid proportion obtained in each culture condition tested (p = 0.02). (B) Sperm enumeration in prepubertal (2.5 dpp and 6.5 dpp) mice testes cultured during one wave of in vitro spermatogenesis. (B1) Flagellated spermatozoa generated in presence of RE were detected after the dissection of 2.5 dpp old testes cultured during 34 days. Photomicrographs were captured at ×500 (Shorr staining) magnification and the scale bar represents 40 μm. (B2) Mean number of spermatozoa [file pone.0116660.s001.tif]

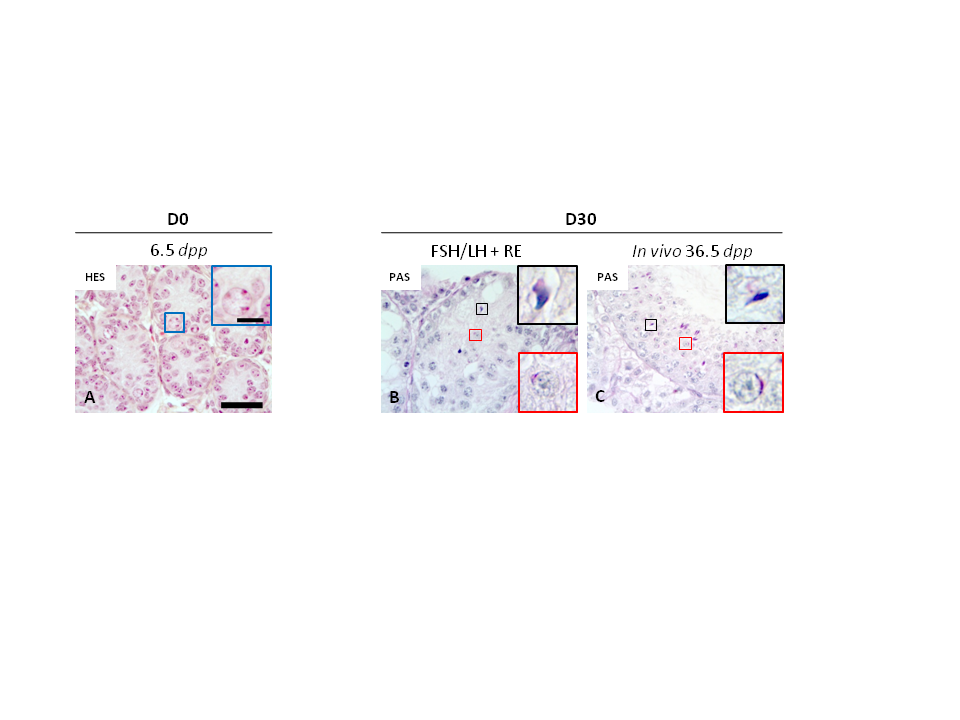

Supplement: S2 Fig — (A) At D0 of culture, spermatogonia (blue box) were the only germ cells present in the seminiferous tubules and are enlarged in the upper inset of the photomicrograph. (B) At D30 of culture, round (red box) and elongated (black box) spermatids were detected when FSH/LH and RE were present in the culture medium. (C) Round and elongated spermatids of the age-matched in vivo (36.5 dpp) are shown with red and black boxes, respectively. Round and elongated spermatids are enlarged in the lower and upper inset of each photomicrograph, respectively. Photomicrographs were captured at ×500 (A to C) and ×1000 (Insets) magnification. The scale bar represents 40 μm or 5 μm in the photomicrograph or inset, respectively, which apply to all other photomicrographs and insets. Footnotes: D: Day, dpp: day post-partum, FSH: Follicle Stimulating Hormone, HES: Hemalun Eosin Saffron, LH: Luteinizing Hormone, PAS: Periodic Acid-Schiff, RE: Retinol (TIF) [file pone.0116660.s002.tif]

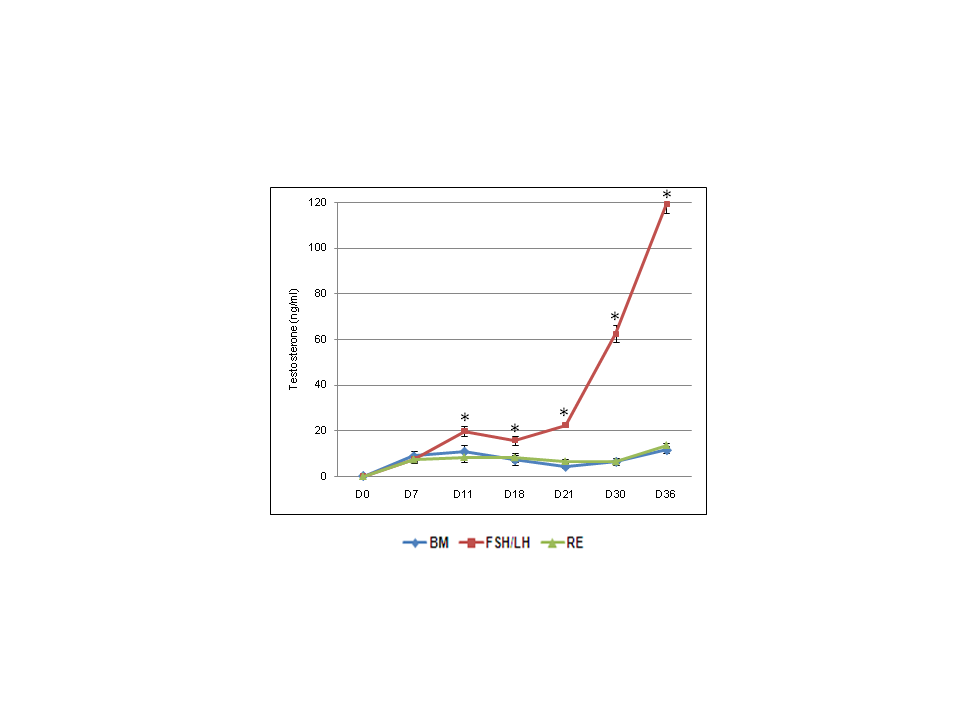

Supplement: S3 Fig — The results are represented as the mean ± s.e.m., n = 4. Asterisk indicates a statistically significant difference between BM and FSH/LH condition (p<0.05). Footnotes: BM: Basal Medium, D: Day, FSH: Follicle Stimulating Hormone, LH: Luteinizing Hormone, n: Number of experiments for each condition, RE: Retinol, s.e.m.: Standard Error of the Mean (TIF) [file pone.0116660.s003.tif]
